# *In Vitro* Murine Leukemia Retroviral Integration and Structure Fluctuation of Target DNA

**DOI:** 10.1371/journal.pone.0031533

**Published:** 2012-02-14

**Authors:** Tatsuaki Tsuruyama, Weizhi Liu, Kenichi Yoshikawa

**Affiliations:** 1 Department of Forensic Medicine and Molecular Pathology, Graduate School of Medicine, Kyoto University, Yoshida-Konoe-cho, Sakyo-ku, Kyoto, Japan; 2 Department of Physics, Graduate School of Science, Kyoto University, Kitashirakawa-cho, Sakyo-ku, Kyoto, Japan; Duke University Medical Center, United States of America

## Abstract

Integration of the retroviral genome into host DNA is a critical step in the life cycle of a retrovirus. Although assays for *in vitro* integration have been developed, the actual DNA sequences targeted by murine leukemia retrovirus (MLV) during *in vitro* reproduction are unknown. While previous studies used artificial target sequences, we developed an assay using target DNA sequences from common MLV integration sites in *Stat5a* and *c-myc* in the genome of murine lymphomas and successfully integrated MLV into the target DNA *in vitro*. We calculated the free energy change during folding of the target sequence DNA and found a close correlation between the calculated free energy change and the number of integrations. Indeed, the integrations closely correlated with fluctuation of the structure of the target DNA segment. These data suggest that the fluctuation may generate a DNA structure favorable for *in vitro* integration into the target DNA. The approach described here can provide data on the biochemical properties of the integration reaction to which the target DNA structure may contribute.

## Introduction

Retroviruses are powerful tools for integrating foreign genes into a host genome. For example, murine leukemia retrovirus (MLV) vectors have been used in the development of induced pluripotent stem cells [1]. However, accidental integration into a host oncogene can induce unexpected transformation due to upregulation of the gene, an effect that is clearly problematic for patients who received gene therapy using an MLV-based vector [2], as well as for gene function analysis. Although integration events have long been considered to occur randomly within the host genome, several recent findings have shown that integration of MLV and HIV-1 occurs more frequently in actively transcribed genes [3], or in promoter regions [4].

There are several reports of *in vitro* integration assays using the terminal cDNA of a long terminal repeat (LTR) in the proviral genome and nonspecific substrate DNA, such as plasmids [5]–[10]. Katz et al. and Kitamura et al. reported on the relative frequency of the use of specific bases as targets for the avian leukosis virus in an *in vitro* integration system [6], [7]. They found that there is a distinct and reproducible pattern of frequently targeted integration sites. The observed specificity is conferred by interaction between integrase and the targets, although the specificity of target integration may be modified by other viral and/or cellular components. In assessing *in vitro* integration, cell extracts have been used to identify the integration sites within the DNA of plasmids such as pUC119, pCG8, pCG14, and pCG28 [5]–[10]. The integration sites were identified using a primer in a proviral genome and plasmid DNAs. Bor et al demonstrated that joining reaction steps promotes bending of the substrate DNA [8]. The preferred sites are adjacent to the loops in the cruciform and are strand-specific. They suggested that the observed preference is due to the end-like character of the stem loop structure, which allows for DNA unpairing. Indeed, previous statistical studies have also demonstrated that weak palindromic sequences are common features of the sites targeted by retroviruses for integration [5], and these palindromic sequences may be associated with the generation of hairpin structures [6]. Despite the attention given to *in vitro* integration systems, their biological significance has not been sufficiently elucidated because existing reports lack comparisons of *in vitro* and *in vivo* integration sites.

In contrast to previous reports, we identified the actual DNA sequence of genes into which MLV commonly integrates. The sequence of *Stat5* has been registered as the target DNA for MLV integration (GENE BANK, DD323316.1) [11]–[14]. In addition, *c-myc* promoter sequence DNA is known to be one of the common integration sites [15]–[19]. In the present study, we report the discovery of unique motifs in the *Stat5* and the *c-myc* promoter sequences which allow for the generation of cruciform structure.

In the present study, only recombinant MLV retroviral integrase, a buffer containing only MgCl_2_, inorganic low-molecular-weight molecules, and host DNA were used in the *in vitro* integration assay. An *in vitro* integration reaction system as simple as that reported here has never been described before, and as such it provides a tool that will yield novel and significant insights into the biochemistry of integration.

## Results

### *In vitro* integration reaction using the *Stat5a*-origin sequence

**Figure 1:**
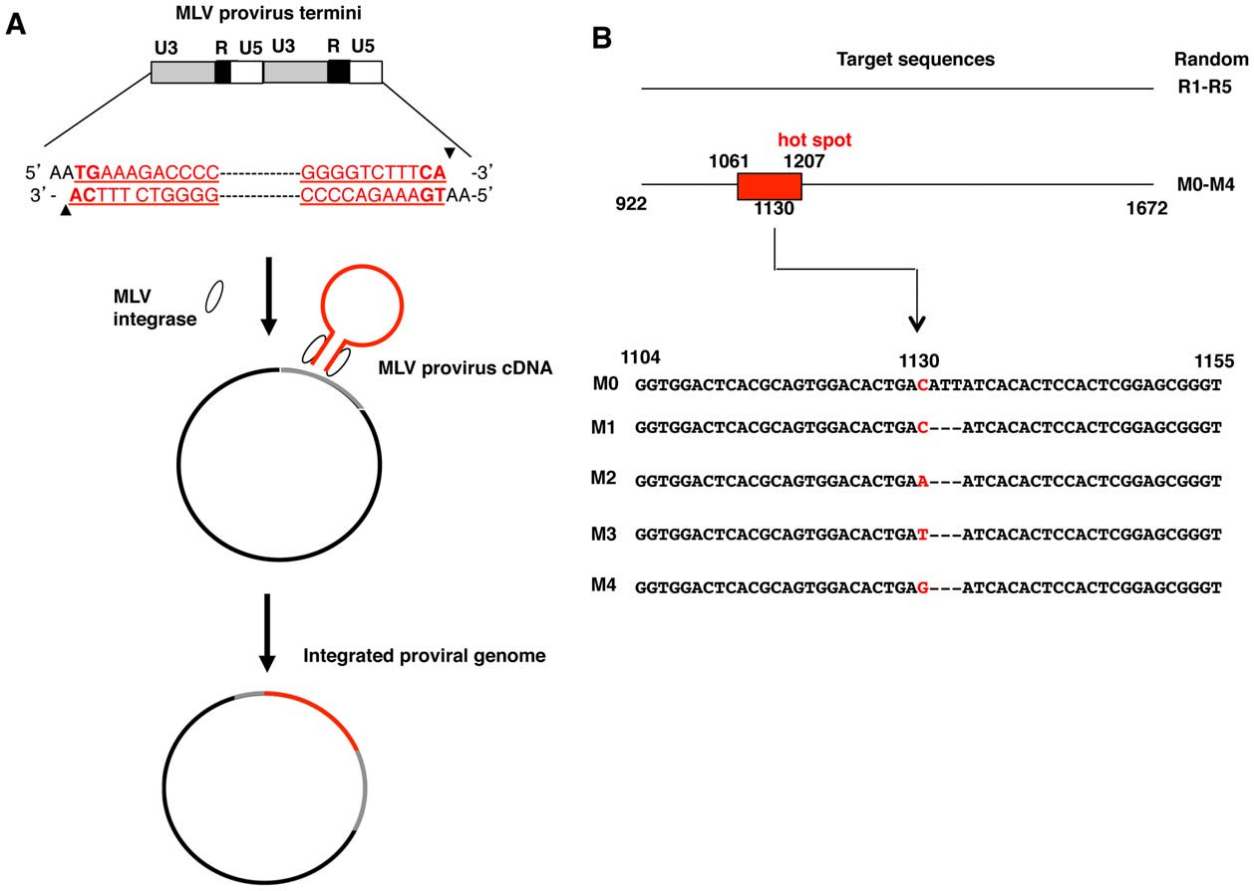
*In vitro* integration using retroviral LTRs. (A) The 5′ and 3′ LTR of MLV proviral DNA (red line) was used after removal of other elements encoding *gag*, *pol*, *pro* and *env*. The sequence shown displays the MLV LTR in the form integrated into the host DNA. The target DNA (grey line) was ligated into the pCR2.1 TOPO plasmid vector (black line). Arrowheads next to the proviral DNA sequence represent the processed ends. After incubation of retroviral and target DNA with integrase, proviral DNA was integrated into the target sequence or plasmid. The integration site was then sequenced. (B) The MLV integration site hot spot in the lymphoma genome of SL/Kh mice is represented by the red square. The utilized target sequences M0-M4 are shown below. M0 is identical to the native *Stat5a* sequence. Red letters in the sequence indicate the most frequent sites of integration in hematopoietic tumors as previously reported by us [14].

We devised an *in vitro* integration assay using a tandem repeat of a long terminal repeat sequence DNA and reaction buffer consisting of 60 mM MgCl_2_ without any other provirus elements. Sites of integration into target sequence DNAs were identified by sequencing the plasmid containing the inserted MLV proviral DNA (Figure 1A). The host DNA originated from the *Stat5a* gene (M0) (No. 922-1322). We modified the target DNA sequence by removing the ATT oligonucleotide (M1) or by replacing the most frequent integration site, cytosine 1130 (M2-4) [12], [14] (Figure 1A). As controls, we used random sequences of 400 bp in length, R1-R5.

### Identification of integration sites

**Figure 2:**
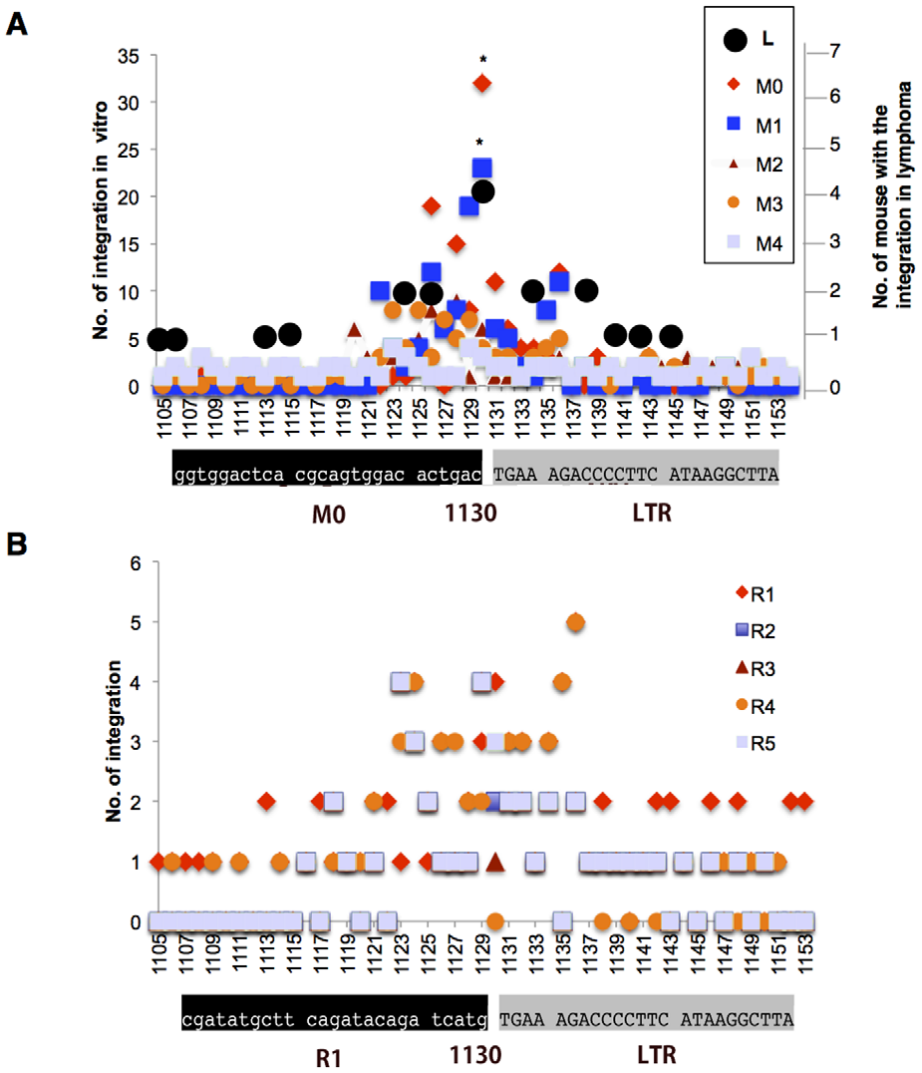
*In vitro* MLV-LTR integration into *Stat5a*. The vertical axis to the left represents the number of integrations into each nucleotide in M0 (native *Stat5a*), modified sequences M1-M4, and control same length random sequences R1-R5. These sequences are 400-bp in length, portions of which are shown in Figure 1. The horizontal axis represents the bases 1105–1153 in the *Stat5a* gene. The sequences shown are the junction of the target sequence and 5′- MLV LTR when the MLV is inserted at nucleotide 1130. (A) Integration sites identified with the *in vitro* assay using sequences M0-M4. Black circles (L) represent the number of mice suffering from lymphomas resulting from MLV integration into the individual nucleotides shown. The number of integrations into nucleotide 1130 per 2000 integrations was significantly greater with sequences M0 and M1 than with sequences M2-M5 (*P<0.05). (B) Integration sites identified with the *in vitro* assay using the 5 random sequences (R1-R5) inserted into the plasmid DNA.

*In vitro* integration sites were identified within the target sequence DNA. The number of integrations into each individual position over approximately 2,000 integrations is shown in Figures 2A and 2B. The number of integrations at cytosine No. 1130 in the M0 and M1 sequences was significantly greater than the number in the M2-M4 sequences and the random sequences R1-R5. These data indicate that integration at cytosine 1130 is affected by replacing the nucleotide but not by removal of the flanking ATT oligonucleotide in the M1 sequence. Significantly, in the region encompassing nucleotides 1124–1140, the frequent locus of MLV integration in spontaneous lymphoma in SL/Kh mice [12], [14], was also found to be a hot spot for *in vitro* integration (L, Figure 2A). A summary of the lymphoma profiles is shown in Table 1. In these lymphomas, expression of *Stat5a* was commonly upregulated.

**Table 1:** Profiles of lymphomas consequent to MLV integration into the genome of pre-B cells in the bone marrow.

Integration site (nucleotide)	No. of mice	*Stat5a*	IgM	Lambda5	VpreB
1105	1	+	+	+	+
1106	1	+	+	+	+
1113	1	+	+	+	+
1115	1	+	+	+	+
1124	2	++	+	+	+
1126	2	++	+	+	+
1130	4	++	+	+	+
1134	2	++	+	+	+
1138	2	+	+	+	+
1140	1	+	+	+	+
1142	1	+	+	+	+
1144	1	+	+	+	+
Total	19				

+, ++: Five- and ten-fold increase in expression level, respectively, relative to normal control pre-B cells in the bone marrow, according to a quantitative RT-PCR assay for *Stat5a* and flow cytometry for IgM, Lambda5, and Vpreb.

### A secondary DNA structure model for the enhancement of integration

**Figure 3:**
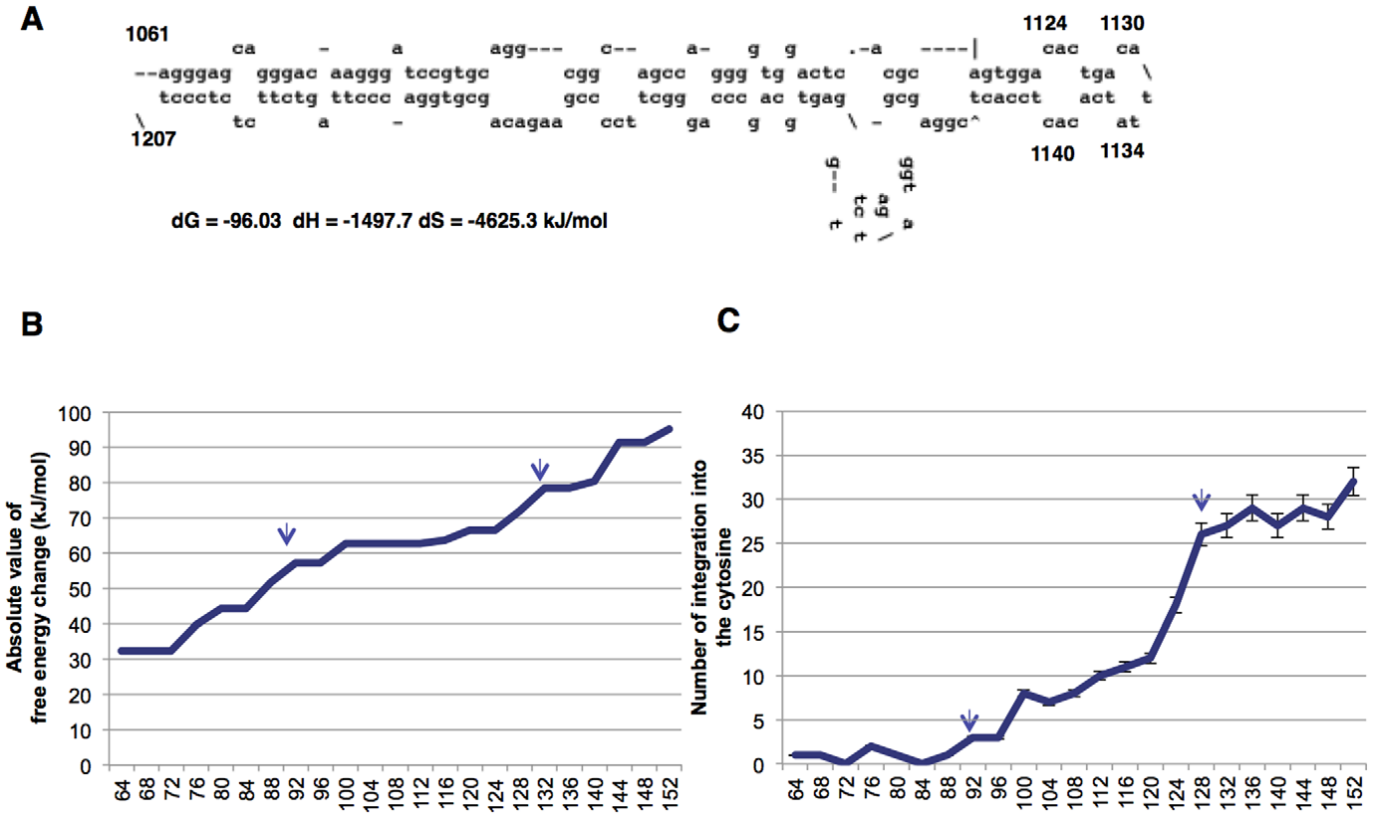
Target sequence length and integration. (A) Presumed secondary structure of the top strand generating a cruciform in the presence of 60 mM of MgCl_2_, as predicted using the M-fold program [20]. (B) Absolute value of the Gibbs' free energy change during DNA folding and generation of a cruciform by the top strand. Arrows represent the marginal points in which the lengths are threshold values of the free energy change. (C) Number of integrations into nucleotide 1130 in the top strand cruciform. Arrows represent the marginal points in which the lengths are the threshold value. Arrows correspond well to the positions of those in (B) (n = 6; mean ± s.d.). The square of the correlation coefficient for the absolute energy value shown in (B) and the number of integrations shown in (C) was 0.838.

We hypothesized that the generation of a secondary structure within the target sequence DNA could explain the observed integration into the segment encompassing nucleotides *Stat5a* 1124–1140. In our proposed model, the free energy change ΔG) in folding resulting when the target DNA focally generates a secondary structure following rewinding after breakage of the complementary binding between dsDNA can be calculated using the M-fold program (http://mfold.rna.albany.edu/?q=mfold/DNA-Folding-Form) [20]. The presumed top strand within the cruciform structure is shown in Figure 3A. Such a cruciform structure for integration was first predicted by Katz [6]. Nucleotides 1124–1140 are located at the top of the secondary structure. Indeed, we calculated the free energy change in the folding target DNA in a stepwise fashion involving 2-base shortenings of the nucleotide 1061–1207 target segment's 5′- and 3′-termini (Figure 3B). As the results demonstrate, when the absolute value of the free energy change decreases to 80 kJ/mol and 60 kJ/mol, the number of integrations into the cytosine position (i.e., near the top of the cruciform structure) decreases significantly. In addition, a close correlation was found between this free energy change and the number of integrations (Figure 3C). These data suggest that the integration process depends on the target DNA structure.

### Structure fluctuation and integration model

**Figure 4:**
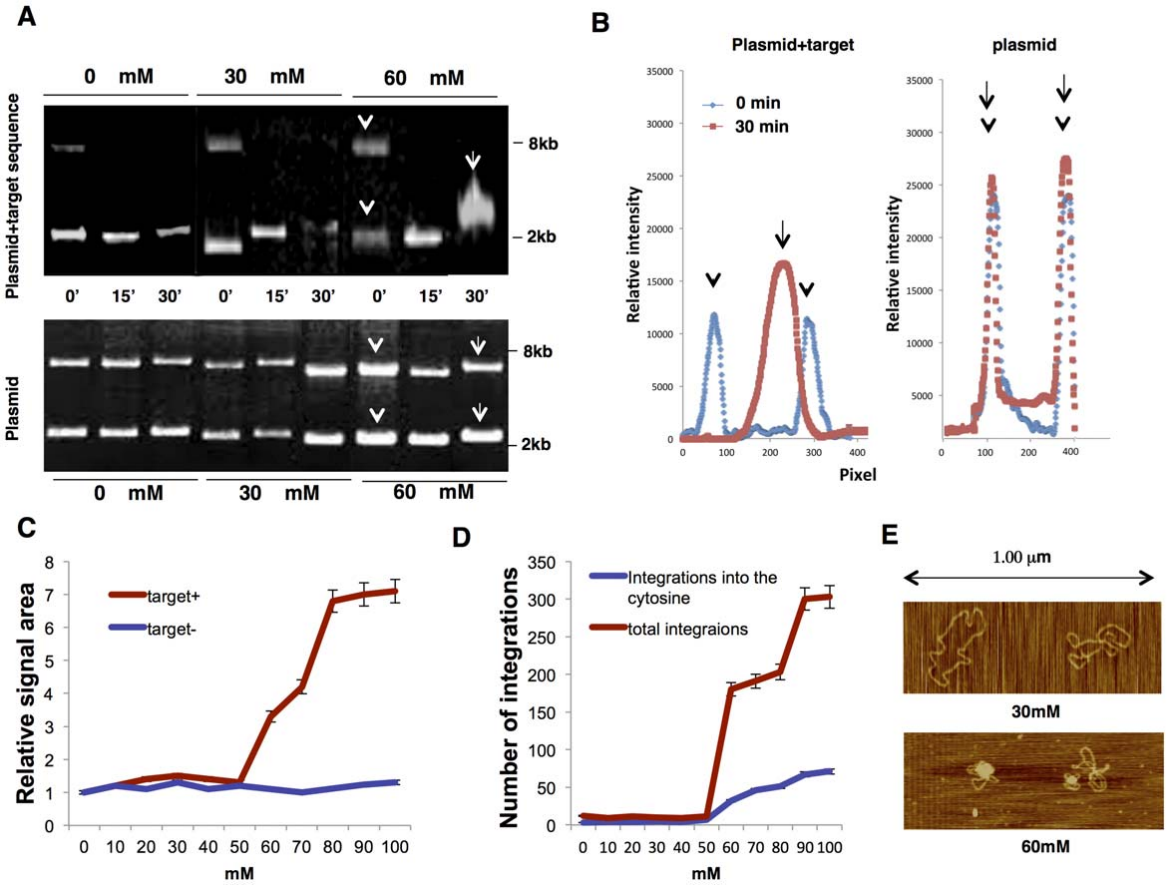
*In vitro* integration in buffers of varying MgCl_2_ concentration. (A) Electrophoresis of plasmid DNA with (upper) or without (lower) the target sequence DNA. Supercoiled DNA was electrophoresed at 0 min, at 30 min, and at 60 min after incubation. Arrows and arrowheads indicate fragments corresponding to peaks shown in (B). (B) Electropherogram of the plasmid with the target sequence and the plasmid alone at incubation for 0 min and 30 min. (C) Graph showing the relative area of the electrophoretic signal of supercoiled plasmid DNA with (red) or without (blue) target *Stat5a* DNA at 60 min after incubation in buffer containing various concentrations of MgCl_2_ (unit mM, n = 6; mean ± s.d.). (D) Graph showing the total number of integration sites within the target *Stat5a* DNA (red) and the number of integrations at nucleotide 1130 (blue) at 60 min after incubation in buffer containing various concentrations of MgCl_2_ (unit mM, n = 6; mean ± s.d.). (E) Sample photo of a secondary structure when using a buffer containing 60 mM and 30 mM of manganese dichloride (mM). Supercoiled DNA (in the upper photo) and globular DNA (in the lower photo) are displayed.

We subsequently evaluated the possibility of target DNA folding. Because no macromolecular components other than DNA and recombinant integrase are utilized in our assay, generation of the secondary structure is probably induced by structure fluctuation. We evaluated the potential for structural fluctuation by comparing plasmids with and without the target DNA using electrophoresis. During incubation of the target DNA in the reaction buffer for 0 to 60 minutes, we electrophoresed the plasmid. The supercoiled plasmid was observed near the level of the 2.5 kb molecular marker (Figure 4A). The signal associated with the supercoiled plasmid DNA was measured by determining the area of the electropherogram peaks (Figure 4B), and this analysis indicated that there is a threshold MgCl_2_ concentration with a respect to fluctuation near 60 mM (Figure 4C). This fluctuation was evident in the electrophoretic migration of the plasmid including the target sequence but not in that of the empty control plasmid. No such threshold concentration of MgCl_2_ was detected in analyses involving linear DNA strands (data not shown). The fluctuation was therefore attributed to the supercoiled or secondary structure of the plasmid. In parallel with this, we found that the number of integrations at the nucleotide 1130 position increased significantly when the MgCl_2_ exceeded 60 mM. Indeed, the number of integrations at this position is closely correlated with fluctuation of the supercoiled DNA structure (Figure 4D). By using an atomic force microscopy, we observed supercoiled plasmid DNA including target DNA in buffer containing 30 mM and 60 mM of MgCl_2_ (Figure 4E). The ratio of supercoiled DNA was significantly higher in the buffer containing 30 mM of MgCl_2_ (82.3% in 30 mM vs. 5.6% in 60 mM, p<0.001); in contrast, the ratio of intersected globule DNA was significantly higher in the buffer containing 60 mM of MgCl_2_ (17.7% vs. 94.4%, p<0.001). Therefore, we assumed that the structure of the target DNA affects integration at nucleotide 1130.

### *In vitro* MLV assay using *c-myc* promoter origin sequence

**Figure 5:**
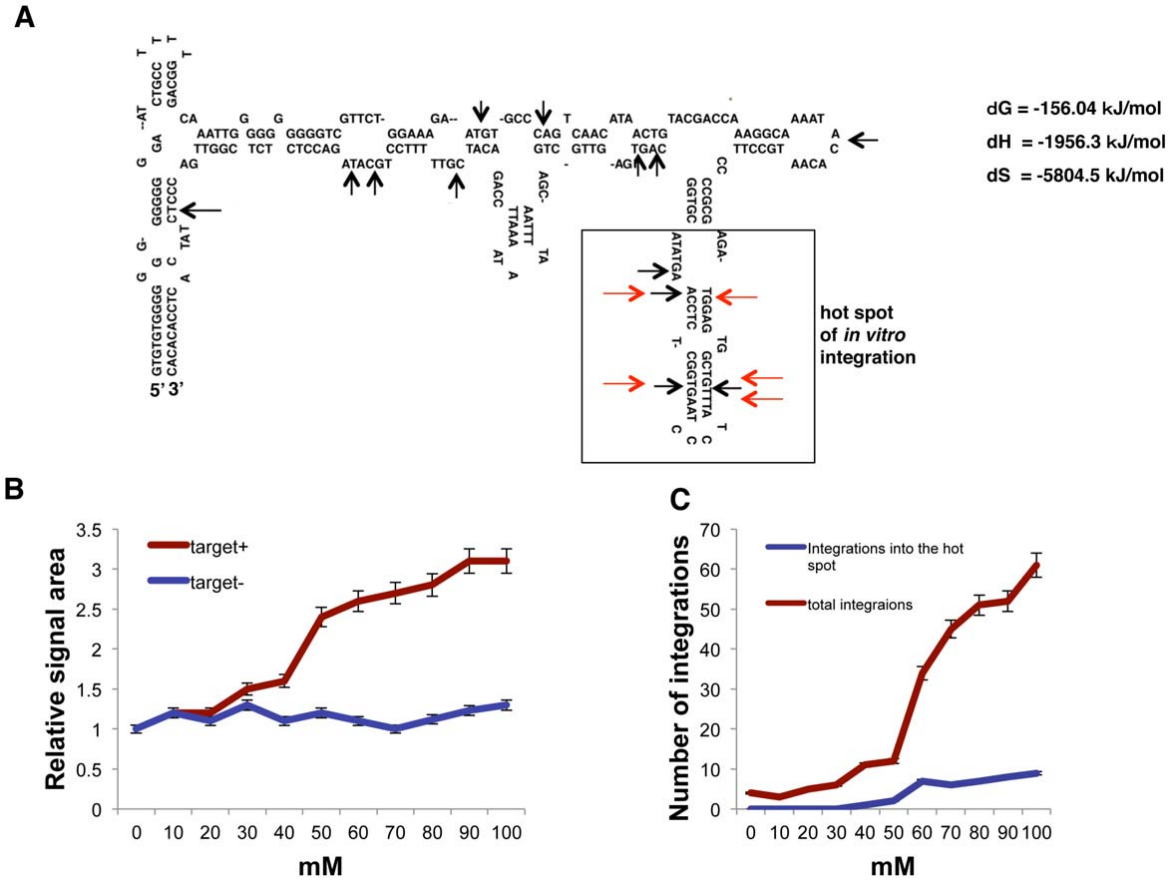
*In vitro* integration using the *c-myc* promoter sequence and buffers containing variable concentrations of MgCl_2_. (A) Thermodynamic analysis of the presumed cruciform structure from the *c-myc* promoter sequence DNA (GENEBANK M12345, No. 711-980) in the presence of 60 mM MgCl_2_. Black arrows indicate the previously reported integration sites [15]–[19]. Red arrows indicate representative *in vitro* integration sites. A box indicates the hot spot segment. (B) Graph showing the relative area of supercoiled plasmid DNA with (target +) or without target *c-myc* DNA (target −) at 60 min after incubation in buffer containing various concentrations of MgCl_2_ (unit mM, n = 6; mean ± s.d.). (C) Graph showing the total number of integration sites within the target *c-myc* DNA and the number of integration into the hot spot at 60 min after incubation in buffer containing various concentrations of MgCl_2_ (unit mM, n = 6; mean ± s.d.).

In addition, we performed an *in vitro* insertion assay using the *c-myc* promoter sequence (GENEBAKN M12345, 711–980) (Fig. 5A). Remarkably, in *in vitro* integration in a buffer containing 60 mM of MgCl_2_, the *in vitro* insertion sites were converged within the top of a presumed branch of cruciform structure. We named the structure hot spot of in vitro integration (Fig. 5A).

To study whether Mg^2+^ intensity influences the integrase activity itself, we incubated host *c-myc* DNA The electrophoretic DNA signal area of the supercoiled plasmid was measured by electropherogram peaks as aforementioned, and there was a threshold concentration with a respect to fluctuation near 50 mM (Figure 5B).

Next, the numbers of the integration hot pots at which integration occurred more than one time in this current assay were plotted under variable concentrations of MgCl_2_. The result demonstrated that the number of times of integration into the hot spot was significantly increased when MgCl_2_ was beyond 50 mM (Fig. 5C). These data and analyses indicated that there was a critical concentration in *in vitro* integration in using *c-myc* promoter sequence DNA.

## Discussion

The present cell-free assay system allowed for a detailed examination of retroviral integration. We utilized the *Stat5a* and *c-myc* sequences as representative sequences that may generate presumed cruciform structures. Integration bias was observed in *in vitro* integration assays using these sequences. Notably, the integration sites were frequently observed near the top of the loop in the cruciform structure. To explain the integration bias, the thermodynamic approach for anticipation of the secondary structure in target DNA was introduced, as we did in the analysis of HIV-1 *in vitro* integration [21]. In the present article, we analyzed the *Stat5a* target sequence because of its simple structure. Figure 3B&C demonstrate a close correlation between the number of *in vitro* integrations into cytosine at the hot spot and the absolute value of free energy change, indicating that the more stable cruciform structure was more favorable for *in vitro* integration into the No. 1130. In addition, the number of integrations into the cytosine discontinuously changed along the length of the target DNA that determined the free energy change in folding. Thus, we supposed that the integration bias was considerably sensitive to the stability of the DNA structure, and there may be a threshold value of free energy change with respect to the bias.

To enhance fluctuation of the DNA structure, the *in vitro* assessment was performed using variable concentrations of MgCl_2_. Indeed, electrophoresis mobility of the target DNA showed that there was a threshold concentration of MgCl_2_ in the reaction buffer. Beyond 50–60 mM of MgCl_2_, the DNA signal was significantly diffused, suggesting that this concentration was the marginal concentration for DNA structure transition in this system. In parallel, the number of integrations significantly increased. For this reason, the 50–60 mM concentration was evaluated for integration efficiency and integration bias by the number of integrations into the frequent integration sites. Our idea was that several DNA structures favorable for integration, including the cruciform structure, were yielded during the fluctuation. Once generated by fluctuation, such secondary structure is relatively stable, because the contained Mg^2+^ neutralized the negative charge on the DNA strand and stabilized the secondary structure as displayed in Figure 4E [22], [23]. The interactions of Mg^2+^ and the DNA fragment gave rise to a large structural deformation at the base pair region. We therefore suppose that fluctuation and stability of target DNA structure influences *in vitro* integration.

Our assay system has advantages relative to previously reported cell-free system assays for *in vitro* integration because of the use of actual gene sequences. The high reproducibility of MLV integration into *Stat5a* by *in vitro* integration suggests integration biochemistry in a secondary structure in a target DNA dependent manner.

## Methods

### Mice and lymphoma clones

All mice used in this study were handled in strict accordance with guidelines for proper animal practice defined by the relevant national and local animal welfare bodies. All animal work was preapproved by the Kyoto University Ethics Committee for Animal Experiments. The approval ID for this study is Med Kyo 09082. The SL/Kh mice were obtained from the RIKEN Bioresource Center, Tokyo, Japan [12], [14]. Flow cytometry analysis of lymphoma tissue was carried out as previously described [12], [14].

### FACS analysis and RT-PCR for lymphoma profiling

The detailed protocol was described previously [12], [14], [24].

### Preparation of MLV integrase

Full-length murine integrase cDNA was obtained from an AKR inbred mouse (Shimizu, Tokyo, Japan) and subcloned into cloning sites I (*Eco*RI) and II (*Xho*I) of the transfer vector, pSYNGCH (Katakura Industries, Saitama, Japan). The procedures for constructing recombinant virus and viral infection of *Bombyx mori* larvae have been reported elsewhere [25]. To construct the recombinant baculovirus, a monolayer of BmN cells (2×10^6^ cells/ml) was cotransfected using 0.5 mg of Abv baculovirus DNA (a linearized AcNPV-BmNPV hybrid-type baculovirus DNA (Katakura, Maebashi, Japan)) and 1.0 mg of psYNGCH-Th integrase in the presence of Insectin liposomes (Invitrogen, Carlsbad, CA, USA). The cotransfected BmN cells were cultured at 27°C for 5 days, after which silkworm pupae were infected with the culture supernatant and then harvested after 6 days. Pupae were suspended in 30 ml of ice-cold homogenizing buffer A (20 mM Tris-HCl, 150 mM NaCl, 1 mM EDTA, 1 mM EGTA, 1 mM DTT, 0.05% phenylthiourea, pH 8.0) containing a protease inhibitor mixture (1 mM phenylmethanesulfonyl fluoride, 10 mM benzamidine), and were disrupted for 5 min in a homogenizer set at 3,000 rpm. The homogenate was centrifuged at 500×*g* for 1 h at 4°C. After removal of the supernatant, the pellet was suspended in 30 ml of homogenizing buffer B (20 mM Tris-HCl, 150 mM NaCl, 1 mM EDTA, 1 mM EGTA, 1 mM DTT, 0.05% phenylthiourea, pH 8.0) containing protease inhibitor mixture, then mixed in a Dounce Teflon homogenizer on ice at 1,000 rpm for 10 strokes. The pellet was resuspended in buffer containing EDTA and DTT (final concentration of 10 mM each), then solubilized with the sulfobetaine detergent Zwittergent 3–12 (Calbiochem, San Diego, CA, USA). The final concentration of detergent was 2% (w/v). The sample was stirred gently for 1 h at 4°C, then centrifuged at 500×*g* in a Hitachi RP50-2 rotor for 1 h at 4°C. The supernatant was collected, and integrase was purified by column chromatography using the His-tag expressed on the integrase. The integrase-containing fractions were pooled and dialyzed against 20 mM HEPES (pH 7.6), 1 M NaCl, 1 mM EDTA, and DTT-20% (w/v) glycerol. The integrase was purified further by dialysis against 20 mM HEPES (pH 7.6), 0.4 M potassium glutamate, 0.1 mM EDTA, 1 mM DTT-0.1% (v/v) Nonie P-40, 20% (w/v) glycerol, which precipitated the integrase. The resulting suspension was centrifuged at 12,500×*g* for 25 min and the pellet was resuspended in 20 mM HEPES (pH 7.6), 1 M NaCl, 1 mM EDTA, 1 mM DTT-10% (w/v) glycerol. This mixture was incubated at 4°C for 30 min and then centrifuged at 12,500×*g* for 25 min. The resulting pellet was resuspended in 20 mM HEPES (pH 7.6), 1 M NaCl, 1 mM EDTA, 1 mM DTT-10% (w/v) glycerol. This mixture was incubated for 30 min at 4°C and then centrifuged at 12,500×*g* for 25 min. The supernatant, which contained the soluble integrase, was collected after centrifugation.

### *In vitro* integration

Target sequence DNA ligated to pCR2.1 plasmid DNA (Invitrogen, Carlsberg, CA) was utilized as the substrate DNA. Briefly, the reaction buffer contained varying concentrations of MgCl_2_ (10 mM to 100 mM), 80 mM potassium glutamate, 10 mM mercaptoethanol, 10% DMSO, and 35 mM MOPS (pH 7.2). First, 150 ng of tandem repeat from MLV LTR cDNA and 200 ng of substrate DNA were incubated with 50 ng of recombinant MLV integrase in 10 µl of binding buffer for 1 h at 30°C. After the reaction, plasmids including a target sequence segment or a random sequence segment were independently transfected into *E. coli* (Invitrogen), after which the plasmid DNAs were extracted. Plasmids with the MLV LTR insertion were sequenced using an Applied Biosystem 3500 DNA sequencer to identify the integration sites.

### Electropherogram analysis

The intensity of electrophoresed DNA bands was stained by ethylene bromide and was measured using a BAS-2000 (Fuji film, Tokyo, Japan). Multi-gauge software was used to integrate the signals from 0 to 400 pixels.

### Atomic force microscopy

DNA containing 10 mM spermidine was then placed on a freshly cleaved piece of mica (30–50 mm). Spermidine was used to aid in the adsorption of DNA molecules onto the mica surface. After 5 min, the sample droplet on the mica was washed with water and dried with N_2_ gas. The DNA molecules were analyzed under the tapping mode on an atomic force microscope (NVB100, Olympus Optical Co., Ltd., Tokyo, Japan; AFM controller and software: Nanoscope IIIa, Digital Instruments, Veeco, Camerillo, CA) in air at room temperature.

### Statistical analysis

Unpaired *t*-tests were performed using SPSS software (SPSS, Chicago, IL, USA), and *P* values<0.01 were considered to indicate statistical significance.
